# CircMAP3K5 promotes cardiomyocyte apoptosis in diabetic cardiomyopathy by regulating miR‐22‐3p/DAPK2 Axis

**DOI:** 10.1111/1753-0407.13471

**Published:** 2023-09-21

**Authors:** Ming Shen, Yuanbin Wu, Libing Li, Liyue Zhang, Gang Liu, Rong Wang

**Affiliations:** ^1^ Department of Cardiovascular Surgery The First Medical Center of PLA General Hospital Beijing China; ^2^ Department of Cardiology The First Hospital of Hebei Medical University Shijiazhuang China; ^3^ Department of Emergency The Seventh Medical Center of PLA General Hospital Beijing China

**Keywords:** apoptosis, circular RNA, DAPK2, diabetic cardiomyopathy, miR‐22‐3p

## Abstract

**Background:**

Diabetic cardiomyopathy (DCM) is one of the serious complications of the accumulated cardiovascular system in the long course of diabetes. To date, there is no effective treatment available for DCM. Circular RNA (circRNA) is a novel r2egulatory RNA that participates in a variety of cardiac pathological processes. However, the regulatory role of circular RNA MAP3K5 (circMAP3K5) in DCM is largely unclear.

**Methods and Results:**

Microarray analysis of DCM rats' heart circular RNAs was performed and the highly species‐conserved circRNA mitogen‐activated protein kinase kinase kinase 5 (circMAP3K5) was identified, which participates in DCM processes. High glucose‐provoked cardiotoxicity leads to the up‐regulation of circMAP3K5, which mechanistically contributes to cardiomyocyte cell death. Also, in high glucose‐induced H9c2 cardiomyocytes, the level of apoptosis was significantly increased, as well as the expression of circMAP3K5. In contrast, the depletion of circMAP3K5 could reduce high glucose‐induced apoptosis in cardiomyocytes. In terms of mechanism, circMAP3K5 acts as a miR‐22‐3p sponge and miR‐22‐3p directly target death‐associated protein kinase 2 (DAPK2) in H9c2 cardiomyocytes, where in circMAP3K5 upregulates DAPK2 expression by targeting miR‐22‐3p. Moreover, we also found that miR‐22‐3p inhibitor and pcDNA DAPK2 could antagonize the protective effects brought by the depletion of circMAP3K5.

**Conclusion:**

CircMAP3K5 is a highly conserved noncoding RNA that is upregulated during DCM process. We concluded that circMAP3K5 promotes high glucose‐induced cardiomyocyte apoptosis by regulating the miR‐22‐3p/DAPK2 axis. The results of this study highlight a novel and translationally important circMAP3K5‐based therapeutic approach for DCM.

## Graphical abstract

Highlights

- Novel mediated cardiomyocyte apoptosis in diabetic cardiomyopathy (DCM) mediated by a new regulator, circRNA mitogen‐activated protein kinase kinase kinase 5 (circMAP3K5).
- New signaling pathways mediating cardiomyocyte apoptosis in DCM.
- CircMAP3K5 promotes cardiomyocyte apoptosis through the miR‐22‐3p‐DAPK2 axis in DCM.

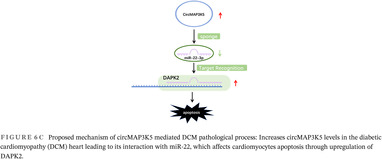

## 1 INTRODUCTION

Diabetes mellitus is a global disease with high morbidity and mortality, which is one of the most important diseases severely harmful to human health.^1^, ^2^ It can cause myocardial microvascular disease and metabolic disorders, which we call diabetic cardiomyopathy (DCM).^3^, ^4^ On this basis, pathological changes such as myocardial microvascular injury and myocardial interstitial fibrosis can occur, leading to cardiac dysfunction, eventually leading to congestive heart failure, cardiogenic shock or arrhythmia, and sudden death in severe cases.^5^ Although the advancement in pharmaceutical treatment and interventional and surgical techniques, it has not yet to find an effective therapeutic method to halt the deterioration of cardiac function due to high levels of blood glucose.^6^ There is an urgent need to deepen the understanding of the pathogenesis and molecular mechanism of DCM to assist the development of novel therapeutic approaches.

Circular RNA (circRNA) is a kind of noncoding RNA molecules with a closed circular structure, without 5′ cap‐structure and 3′ poly(A)‐tail.^7^ CircRNA mainly exists in cytoplasm or exosomes and has the characteristics of tissue specificity, disease specificity, timing specificity, and high stability.^7^ In recent years, a large number of studies have shown that circRNA is closely related to biological growth and development, stress response, disease occurrence and development, and so forth. CircRNA can act as a sponge of microRNA, involving in the regulation of disease‐related genes in the transcriptional level and post‐transcriptional level through a competing endogenous RNA mechanism.^8^ Although increasing evidence has been found to reveal the function of circRNA in biological processes, especially in various kinds of diseased conditions, there is still a long way to go to bridge the knowledge gap. Recently, a series of studies reveal the association between circRNA and cardiovascular disease. For example, Garikipat reported that circular RNA CircFndc3b actively participated in cardiac repair after myocardial infarction through regulation of FUS/VEGF‐A axis.^9^ What is more, circRNAs are of prime importance in heart failure. Hsa_circ_0097435 is overexpressed in heart failure patients and can sponge multiple microRNAs to accelerate the progression of heart failure. To develop a new therapeutic method, hsa_circ_0097435 can be a promising target.^10^ Additionally, circRNAs play a role in atherosclerosis, coronary artery disease, myocardial infarction, as well as cardiomyopathy.^11^

Here, we investigated novel circRNAs as potential therapeutic targets for DCM. Our circRNA sequencing approach identified a novel, highly abundant, and sequence conserved circRNA molecule derived from the host gene encoding the mitogen‐activated protein kinase kinase kinase 5 (circMAP3K5). We applied various gain‐ and loss‐of‐function studies specific for circMAP3K5 to validate its pathological effects and elucidate the underlying mode of action in vitro. CircMAP3K5 was found to regulate apoptosis of cardiomyocytes in DCM. In addition, it was shown that circMAP3K5 accelerated cardiomyocytes apoptosis through the miR‐22‐3p/death‐associated protein kinase 2 (DAPK2) axis. In this study, we discovered circMAP3K5 as a promising target for the prevention of DCM.

## 2 MATERIALS AND METHODS

### 2.1 Animals and specimens

Twenty male Sprague–Dawley rats, weighted 210–240 gram, were intraperitoneally injected streptozocin (60 mg/kg, Sigma) to induce type 1 diabetes mellitus (T1DM). After 3 days, if fasting blood glucose was greater than 11.1 mmol/L, it is considered to be a successful induction of T1DM model. Eight weeks after modeling, the rats were anesthetized and left ventricular free wall tissues were cut off for isolation of total RNA. The study was approved by the medical ethics committee of Chinese PLA General Hospital.

### 2.2 Total RNA isolation and quality control

Total tissue RNA was extracted from the left ventricular free wall tissue of normal and diabetic Sprague–Dawley rats using TRIzol reagent (Invitrogen, Waltham, MA, USA) according to the manufacturer's directions. RNA quality and concentration were determined using NanoDrop ND‐1000 (Thermo Fisher Scientific, Wilmington, NC, USA). RNA integrity and genomic DNA (gDNA) was measured by electrophoresis on a denaturing agarose gel. Samples were stored at −80°C for further analysis.

### 2.3 circRNA microarray analysis

Sample labeling and array hybridization were performed according to the manufacturer's instrument. Total RNA was digested with RNase R (Epicenter, Inc.) to eliminate linear RNA and enrich for circRNA. These circRNAs were amplified and transcribed into fluorescent cRNA using a random primer method (Arraystar Super RNA Labeling Kit; Arraystar). labeled cRNAs that were hybridized to Arraystar Rat circRNA Array V2.0 (8 × 15 K) with a total of 14 145 circRNA probes on the chip. After cleaning the slides, the arrays were swept using an Agilent G2505C scanner. Agilent feature extraction software (version 11.0.1.1, USA) was utilized to analyze the obtained array images. When comparing two groups of profile differences, the “fold change” between the groups for each circRNA is computed. The statistical significance of the difference may be conveniently estimated by *t* test. circRNAs having fold changes ≥2.0 and *p* values ≤.05 are selected as the significantly differentially expressed.

### 2.4 Cell cultures

H9C2 cell line (rat embryonic cardiomyocytes) was obtained from Shanghai Fuheng Biotechnology Co., Ltd. (China) and cultured in Dulbecco's modified Eagle medium (DMEM; Solarbio, China) supplemented with 10% fetal bovine serum (Gibco, USA), 0.1 mg/mL streptomycin (Solarbio, China) and 100 units/mL penicillin (Solarbio, China). The cultures were incubated at 37°C, under 5% CO_2_. A DCM model was established after 48 h of exposure to high glucose (HG) concentration (4500 mg/L, 25 mM, Solarbio, China), and the control group was established after exposure to medium containing low glucose levels (1000 mg/L, 5.5 mM, Solarbio, China).^11^, ^12^ Si‐circMAP3K5, si‐circMAP3K5‐NC, pLc‐circMAP3K5‐vector, pLc‐circMAP3K5, and pcDNA‐DAPK2‐vectors were obtained from Geneseed Biotech Co., Ltd. (Guangzhou Guangdong, China), whereas miR‐22‐3p mimic, mimic‐NC, miR‐22‐3p inhibitor, and inhibitor‐NC were acquired from RiboBio Co., Ltd. (Guangzhou Guangdong, China).

### 2.5 Quantitative real‐time polymerase chain reaction

Total RNA was extracted from the cultured cells using the TRIzol reagent (Invitrogen, USA), then reverse transcribed to complementary DNA (cDNA) using the ReverTRA Ace qPCR Mastermix with gDNA Remover (Code. No. FSQ‐301, Toyo Spin, Toyo) kit. The cDNA was used for quantitative real time polymerase chain reaction (qRT‐PCR) using SYBR® Green Real‐Time PCR Mastermix (TOYOBO qPK‐201) kit. The reaction mixture comprised SYBR Green (5 μL), primer MIX (F + R, 0.5 μL), ddH_2_O (3.5 μL) and cDNA template (1 μL). The standard controls for micro RNA and messenger RNA (mRNA)/circRNA were U6 and GAPDH, respectively. For each group, qRT‐PCR experiments were conducted in three independent replicates. Specific primer sequences used in the reaction are shown in Supplementary Table S1, circRNA primers are designed to span junction sites.

### 2.6 Western blot analysis

Total proteins were extracted from the cultured cells using the RIPA buffer (Solarbio, China), with protease inhibitor (PMSF, Solarbio, China) added. Protein concentration was measured using a BCA protein quantitation kit (Solarbio, China). Next, equal concentrations of protein samples were separated on a 10% via sodium dodecyl‐sulfate polyacrylamide gel electrophoresis, then transferred to PVDF membranes (Millipore, USA). The membranes were blocked with 5% defatted milk, then incubated overnight at 4°C with the following primary antibodies; DAPK2 (Abcam, USA, 1:3000), cleaved caspase‐3 (Proteintech, USA, 1:1000), Bax (Proteintech, USA, 1:1000), Bcl‐2 (Abcam, USA, 1:3000), and HRP‐GAPDH (Proteintech, USA, 1:1000). The next day, membranes were incubated for 1 h with corresponding secondary antibodies, then analyzed for protein expression on the Tanon chemiluminescent image analysis system. Gray values of the obtained results were quantified using ImageJ software.

### 2.7 Luciferase reporter gene assay

Luciferase reporter gene analysis was performed using a fluorometer (Turner Biosystems instrument, USA). Briefly, fluorescence intensities of 293 T cells transfected with a vector containing a control mimic or miR‐22‐3p mimic, circMAP3K5, circMAP3K5 mutant of miR‐22‐3p binding site, DAPK2, and DAPK2 mutant fragment of a miR‐22‐3p binding site were determined using firefly luciferase as an internal reference.

### 2.8 Flow cytometry

Cells were first cultured for 48 h in HG medium then analyzed for apoptosis. Approximately 2 × 10^5^ cells were seeded into 6‐well plates and apoptotic rate analyzed using the Annexin V‐FITC/PI Apoptosis Detection Kit (Solarbio, China), according to the manufacturer's instructions. Next, approximately 2 × 10^5^ cells were collected, washed with binding buffer, stained at 25°C, then subjected to flow cytometry analysis.

### 2.9 Statistical analysis

Data were statistically analyzed and graphed using GraphPad Prism 8. Comparisons between two groups were performed using an unpaired Student's *t* test, whereas those across multiple groups were conducted using a one‐way analysis of variance. Data followed by *p* < .05 were considered statistically significant.

## 3 RESULTS

### 3.1 Successful establishment of the DCM rat model

**FIGURE 1:**
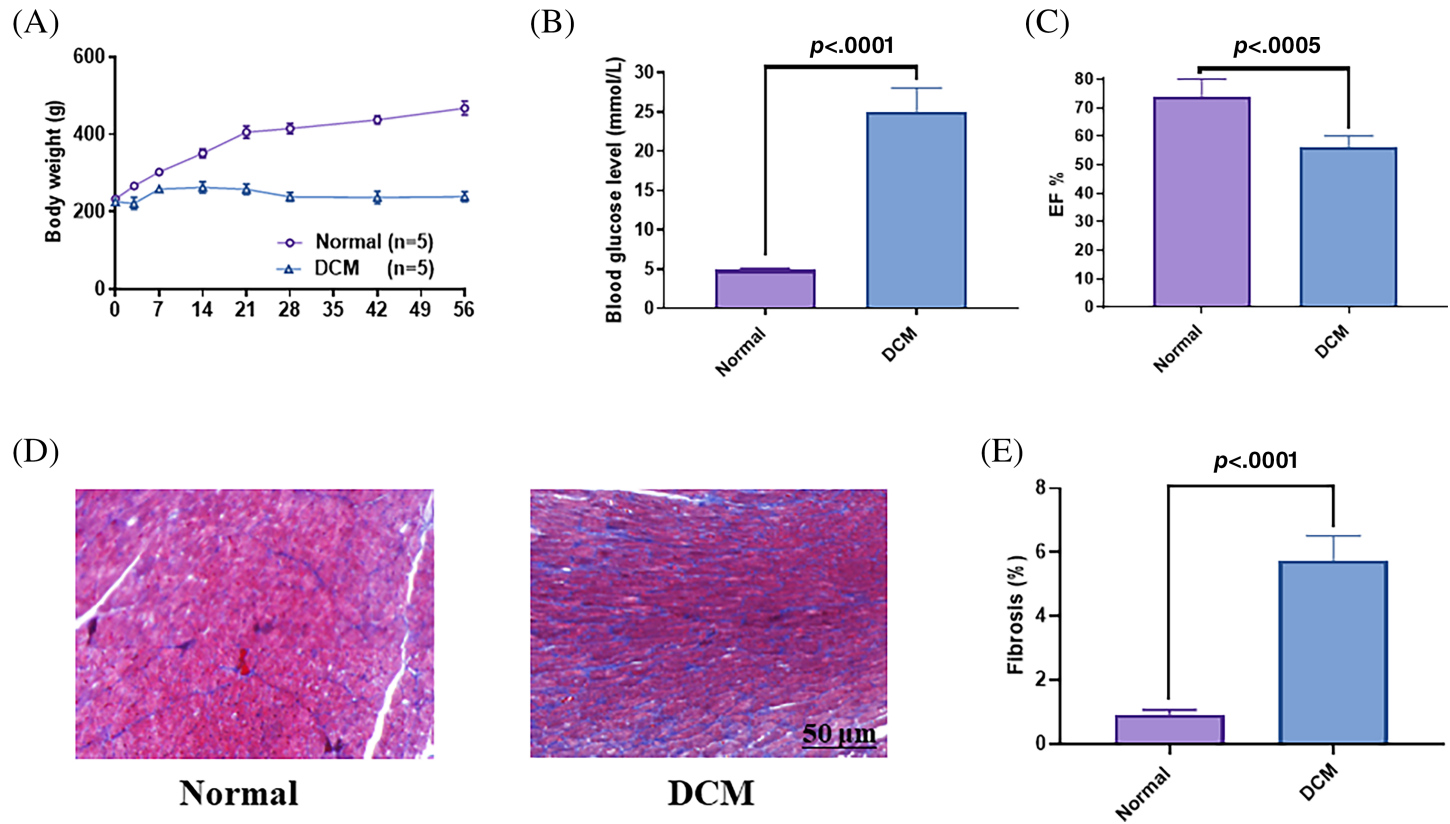
Successful modeling of type 1 diabetic cardiomyopathy. A, Body weight of rats subsequent to establishing the diabetic cardiomyopathy (DCM) model; B, Random blood glucose levels were higher in DCM group (*p* < .0001); C, The DCM group has decreased LVFF values compared with the normal group (*p* =  .0005); D–E, The degree of interstitial fibrosis in DCM animals was greater than that in normal control (*p* < 0.0001). EF, ejection fraction.

After streptozotocin induction, blood sugar level and body weight of rats were measured and documented in a certain time interval. A distinct difference between DCM and normal control rats was observed. In the DCM group, the rat had significantly higher blood sugar levels and lower body weight, which were inconsistent with the typical manifestations and symptoms of diabetes mellitus (Figure 1A,B). This experiment reflects left ventricular systolic function in rats through left ventricular ejection fraction (LVEF) and short‐axis shortening rate. LVEF values were significantly decreased in the DCM group (56.33 ± 1.66%) compared with the normal control group (74.06 ± 2.63%). (Figure 1C). In DCM, the increased deposition of collagen and interstitial fibrosis in the myocardium is one of the characteristic structural changes within it. In this study, LV collagen deposition and fibrosis ratio were markedly increased in DCM rats compared to normal controls (5.74 ± 0.35 vs. 0.94 ± 0.08), using Masson staining method (Figure 1D,E).

### 3.2 Identification and validation of the conserved circular RNA, CircMAP3K5, in DCM

**FIGURE 2:**
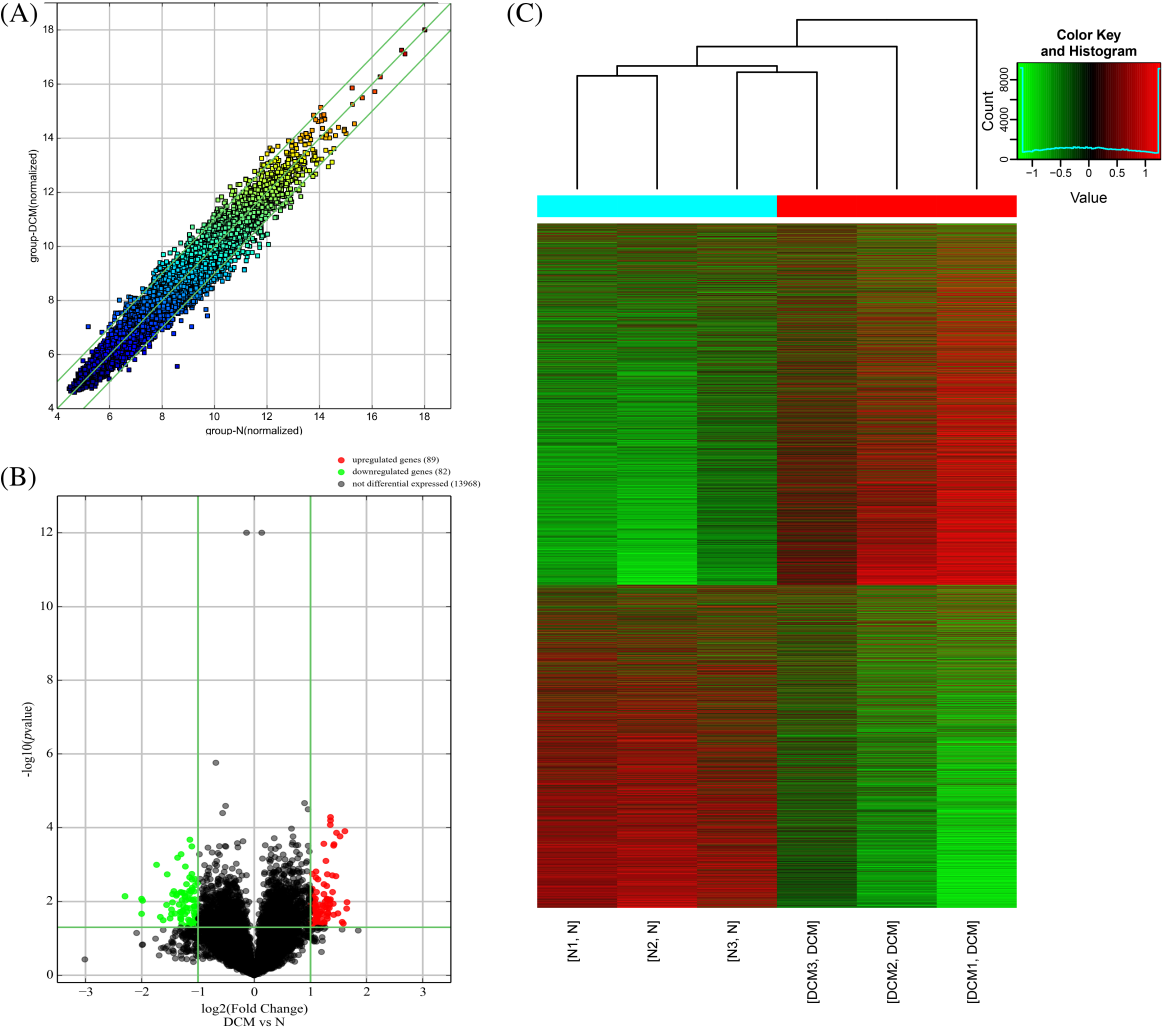
Differential expression of circRNA by A, scatter plot, B, volcanic map, and C, cluster analysis in normal group and diabetic cardiomyopathy (DCM) group. circRNA, circular RNA.

To investigate the circRNA expression profile of DCM, we have applied the Arraystar Rat circRNA Array analysis of specimen from three DCM samples and three normal controls. A total of 14139 circRNAs were detected by Arraystar Rat circRNA Microarray. When comparing two groups of profile differences, the “fold change” between the groups for each circRNA is computed. The statistical significance of the difference is estimated by *t* test. CircRNAs having |fold changes|≥2.0 and *p* values ≤.05 are selected as the significantly differentially expressed. Eventually, 171 differentials expressed circRNAs, including 89 upregulated and 82 downregulated circRNAs were identified. A scatter plot (Figure 2A) and a volcano plot (Figure 2B) showed the distributions of circRNAs more directly. The top 50 dysregulated circRNAs based on fold changes were summarized in Supplementary Table S2. The result of hierarchical clustering showed a distinguishable circRNA expression profiling among samples. The data suggested that the circRNA in DCM was different in normal control samples (Figure 2C). As circRNAs are back spliced from precursor mRNAs, we hypothesized that circRNAs could have comparable function to their host mRNAs. We thus applied gene ontology analyses to pinpoint circRNAs potentially involved in DCM processes. We identified a highly conserved circRNA whose host genes are crucially involved in DCM, namely circMAP3K5. We validated the expression of this circRNA in DCM hearts. The result suggested that circMAP3K5 may have disease significance and be involved in the pathophysiology of DCM.

### 3.3 Depletion of CircMAP3K5 attenuates cardiomyocyte apoptosis in a DCM cell model

**FIGURE 3:**
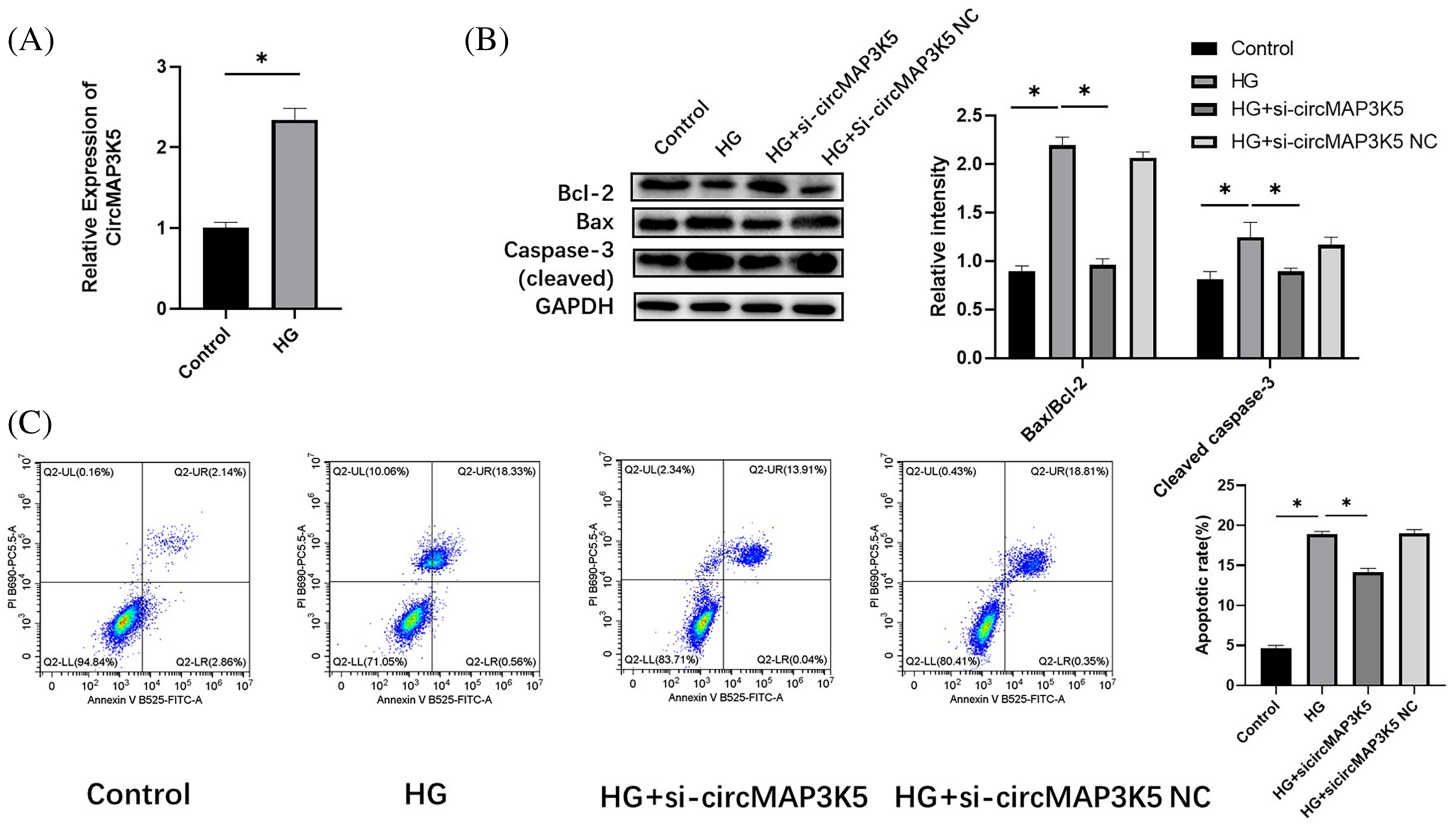
Depletion of circRNA Mitogen‐activated protein kinase kinase kinase 5 (circMAP3K5) attenuates cardiomyocyte apoptosis in a diabetic cardiomyopathy (DCM) cell model. A, quantitative real‐time polymerase chain reaction results show expression levels of circMAP3K5 in samples of DCM (*n* = 3) and control (*n* = 3) cells. B, Western blots showing expression levels of Bcl‐2, Bax, and cleaved caspase‐3 proteins in the samples. C, Flow cytometry results depict the rate of apoptosis. Statistically significant difference: *p < .05 (*n* = 3). circRNA, circular RNA; HG, high glucose.

We investigated the potential relationship between circMAP3K5 and DCM by analyzing levels of circMAP3K5 expression in H9c2 cell models stimulated by a HG environment. Results showed that circMAP3K5 was significantly upregulated in the DCM model relative to the control group (Figure 3A). Moreover, cells treated with a HG concentration exhibited a significantly higher apoptosis rate relative to those under normal culture conditions. However, knocking down circMAP3K5 expression mediated a significant reduction in the rate of apoptosis (Figure 3B,C). Collectively, these results indicated that circMAP3K5 might not only be closely associated with the occurrence and development of DCM but can also directly regulate the rate of apoptosis in cell injury caused by HG. Therefore, we further explored the relationship between circMAP3K5 expression and apoptosis of cardiomyocytes in DCM.

### 3.4 CircMAP3K5 acts as a sponge for miR‐22‐3p in cardiomyocytes

**FIGURE 4:**
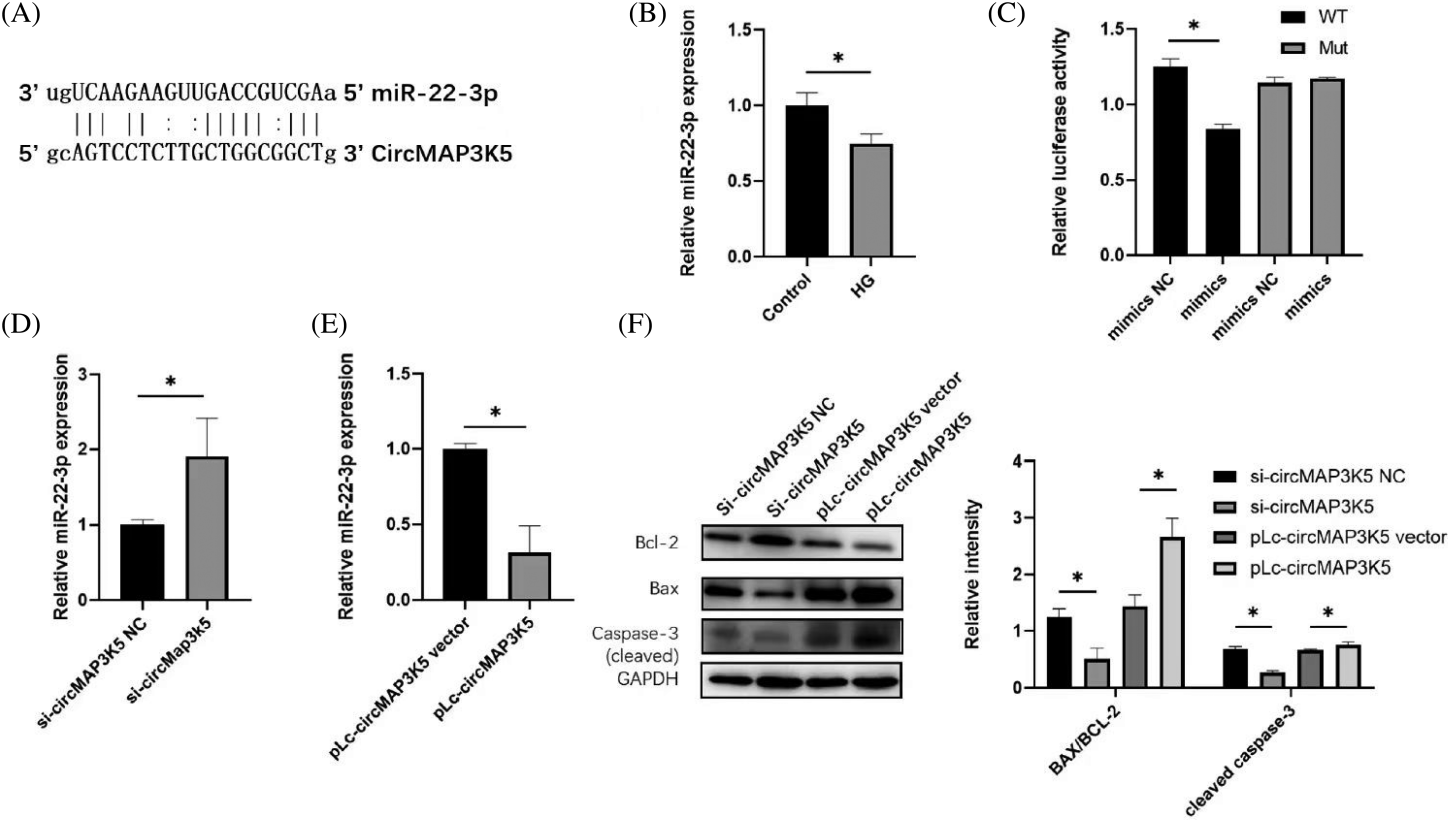
CircRNA mitogen‐activated protein kinase kinase kinase 5 (circMAP3K5) acts as a sponge for miR‐22‐3p in cardiomyocytes. A, Predicted potential interactions between circMAP3K5 and miR‐22‐3p according to Miranda software. B, quantitative real‐time polymerase chain reaction (qRT‐PCR) results showing expression level of miR‐22‐3p in H9c2 cells. C, Luciferase activity in wild‐type circMAP3K5 (circMAP3K5 WT) and a mutant with a miR‐22‐3p binding site (circMAP3K5 MUT) in 293 T cells transfected with a control mimic or a miR‐22‐3p mimic. D, qRT‐PCR results depicting levels of miR‐22‐3p expression in H9c2 cells transfected with either si‐MAP3K5 NC or si‐MAP3K5. E, qRT‐PCR results showing levels of miR‐22‐3p expression in H9c2 cells transfected with either pLc‐MAP3K5 vector or pLc‐MAP3K5. F, Western blots showing expression levels of the apoptosis regulatory protein. Statistically significant difference: *p < .05 (*n* = 3). HG, high glucose; miRNA, microRNA.

Next, we elucidated the potential mechanisms underlying CircMAP3K5‐mediated myocardial injury and DCM‐induced apoptosis in cardiomyocytes. Specifically, we adopted the Miranda software (using default parameters) to predict potential binding sites between CircMAP3K5 and miR‐22‐3p (Figure 4A) and found that DCM downregulated miR‐22‐3p expression in H9c2 cells (Figure 4B). MiR‐22‐3p mimic significantly reduced luciferase activity of wild‐type CircMAP3K5 but did not significantly affect CircMAP3K5 with mutations at the miR‐22‐3p binding site (Figure 4C). In addition, knockdown of CircMAP3K5 significantly upregulated miR‐22‐3p expression in cells (Figure 4D). Conversely, miR‐22‐3p was significantly downregulated in pLcMAP3K5‐transfected cells (Figure 4E). Furthermore, the ratio of pro‐apoptotic gene cleaved caspase‐3 to Bax/Bcl‐2 was down‐regulated in the CircMAP3K5‐knockout cells, whereas an opposite trend was observed in those transfected with pLc‐circMAP3K5 (Figure 4F). Taken together, these results suggested that CircMAP3K5 acts as a sponge for miR‐22‐3p in H9c2 cells.

### 3.5 Direct targeting of DAPK2 to miR‐22‐3p in H9c2 cells

**FIGURE 5:**
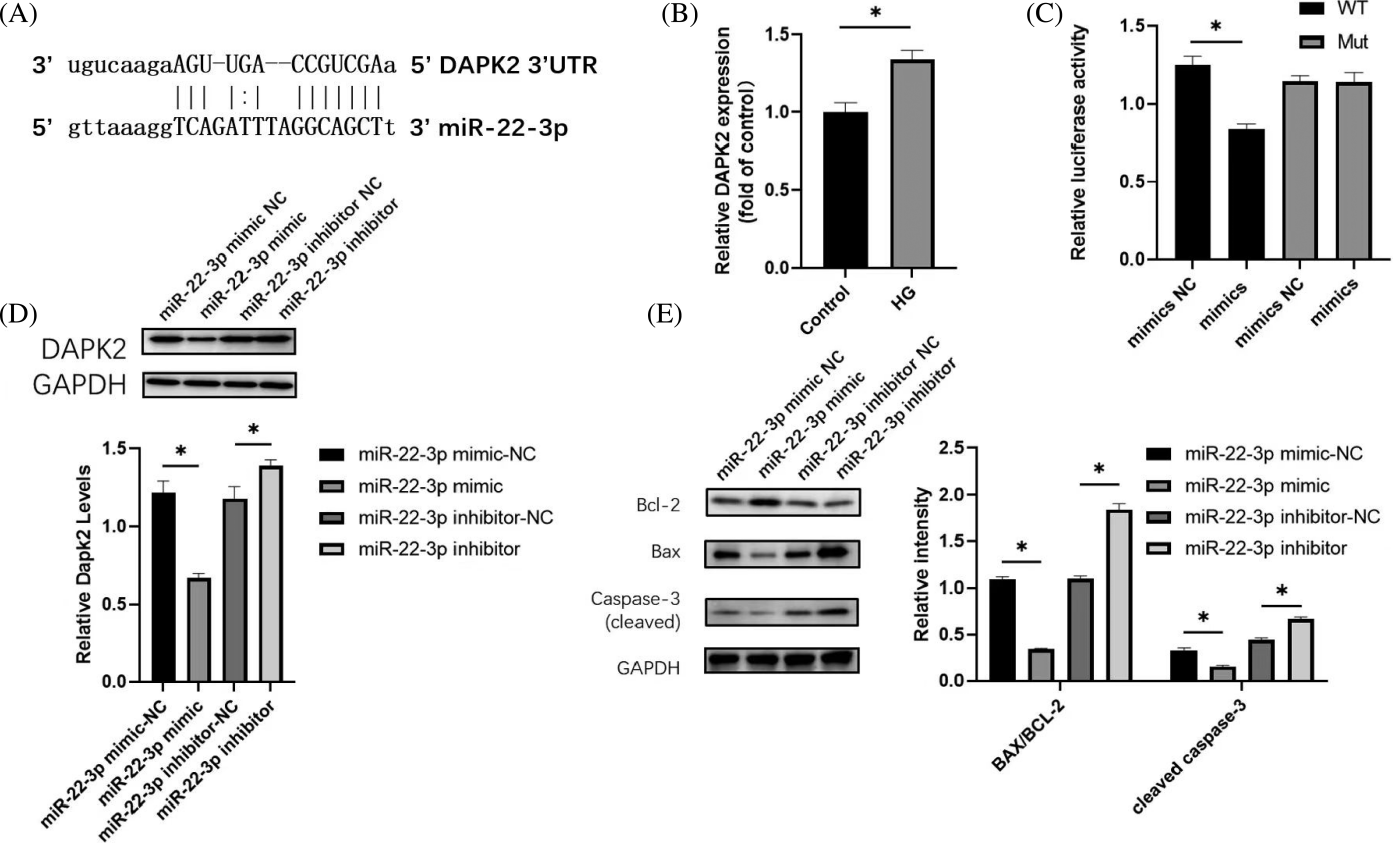
DAPK2 directly targets miR‐22‐3p in H9c2 cells. A. Results from Miranda software showing that DAPK2 is a target gene for miR‐22‐3p. B, qRT‐PCR results depicting levels of DAPK2 expression in HG‐treated H9c2 cells. C, Luciferase activity of wild‐type DAPK2 (DAPK2 WT) and BNIP3 (DAPK2 MUT) with a mutant of the miR‐22‐3p binding site in H9c2 cells transfected with either a control mimic or a miR‐22‐3p mimic. D, Western blots showing expression levels of DAPK2 protein in H9c2 cells treated with miR‐22‐3p mimic NC, miR‐22‐3p mimic or miR‐22‐3p inhibitor NC and miR‐22‐3p inhibitor. E, Western blot showing expression levels of apoptosis regulatory protein. Statistically significant difference: *p < .05 (*n* = 3). DAPK2, death‐associated protein kinase 2; Mut, mutant; qRT‐PCR, quantitative real‐time polymerase chain reaction; WT, wild type.

Next, we adopted Miranda software (using default parameters) to identify miR‐22‐3p's target sites in DAPK2 3′UTR (Figure 5A). Results showed that glucose‐induced damage upregulated DAPK2 expression in H9c2 cells (Figure 5B). Notably, miR‐22‐3p mimic markedly inhibited luciferase activity of wild‐type DAPK2 but did not significantly affect DAPK2 with a miR‐22‐3p binding site mutation in 293 T cells (Figure 5C). Moreover, transfection of miR‐22‐3p mimics significantly downregulated DAPK2 expression, whereas this phenomenon was reversed by miR‐22‐3p inhibitor, further confirming that DAPK2 was reversely regulated by miR‐22‐3p (Figure 5D). In addition, miR‐22‐3p significantly affected the expression of cleaved caspase‐3 and the Bax/Bcl‐2 ratio, with DAPK2 also causing a similar trend. Taken together, these results indicated that miR‐22‐3p affects apoptosis by regulating DAPK2 expression (Figure 5E).

### 3.6 CircMAP3K5 promotes apoptosis in cardiomyocytes by regulating the miR‐22‐3p/DAPK2 axis

**FIGURE 6:**
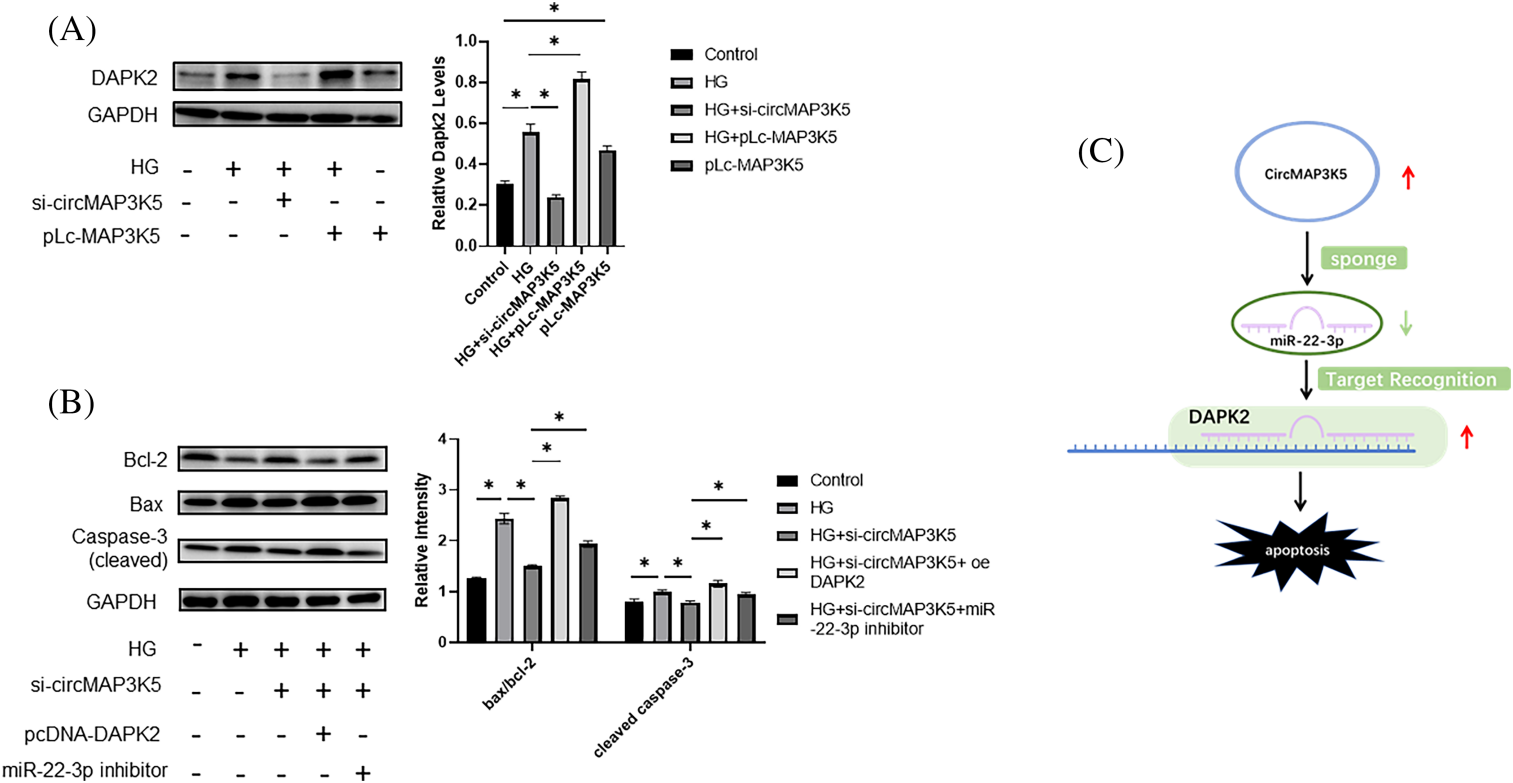
CircRNA mitogen‐activated protein kinase kinase kinase 5 (circMAP3K5) promotes HG‐induced cardiomyocyte apoptosis by regulating the miR‐22‐3p/DAPK2 axis. A, Western blots showing expression levels of DAPK2 protein in cardiac myocytes transfected with either si‐circMAP3K5 or pLcMAP3K5, and exposed to high glucose concentrations (25 mmol/L) for 48 h. B, Western blots showing expression levels of apoptosis‐related proteins in cardiomyocytes transfected with si‐circMAP3K5, miR‐22‐3p inhibitor, and/or pcDNA‐DAPK2, under high glucose concentrations (25 mmol/L). C, Proposed mechanism of circMAP3K5 mediated DCM pathological process: Increases circMAP3K5 levels in the diabetic cardiomyopathy (DCM) heart leading to its interaction with miR‐22, which affects cardiomyocytes apoptosis through upregulation of DAPK2. DAPK2, death‐associated protein kinase 2; HG, high glucose.

Next, we elucidated the role played by the circMAP3K5/miR‐22‐3p/DAPK2 signaling pathway in the apoptosis of cardiomyocytes following HG‐induced cardiomyocyte injury. Results indicated that injury caused by a HG concentration upregulated DAPK2 expression, whereas knockout of CircMAP3K5 significantly reversed this phenomenon in cardiomyocytes under a HG concentration environment. Moreover, transfecting pLcMAP3K5 into cardiomyocytes under normal and HG environments resulted in significant upregulation of DAPK2, indicative of a strong positive correlation between circMAP3K5 and DAPK2 (Figure 6A). At the same time, exposure of H9c2 cells on the HG environment resulted in a high rate of apoptosis, whereas knockdown of CircMAP3K5 effectively reversed this injury. Notably, miR‐22‐3p inhibitor and pcDNA‐DAPK2 reversed the protective effect of circMAP3K5 knockdown in the system (Figure 6B). Taken together, these results indicated that circMAP3K5 promotes HG‐induced apoptosis in cardiomyocytes by regulating the miR‐22‐3p/DAPK2 axis (Figure 6C).

## 4 DISCUSSION

The pathogenesis of DCM, a serious complication of the cardiovascular system that occurs during the long course of diabetes, remains unclear.^13^, ^14^, ^15^ In recent years, numerous studies have described the indispensable role played by circRNAs in many diseases.^16^, ^17^, ^18^, ^19^, ^20^ In this study, we found that the circMAP3K5/miR‐22‐3p/DAPK2 pathway is involved in the occurrence and development of DCM by regulating apoptosis of cardiomyocytes. Results from in vitro experiments showed that circMAP3K5 was overexpressed in cardiomyocytes stimulated by a HG environment. Moreover, overexpressing circMAP3K5 directly inhibited the expression of miR‐22‐3p, thus upregulating that of DAPK2. In addition, silencing circMAP3K5 significantly downregulated DAPK2 expression, by acting as a sponge for endogenous miR‐22‐3p, which in turn inhibited downstream cytokines and suppressed the rate of HG‐induced apoptosis in H9c2 cells. Taken together, our results demonstrated that circMAP3K5 can promote the expression of DAPK2 through miR‐22‐3p and then promote the apoptosis of cardiomyocytes in DCM. Therefore, circMAP3K5 plays an important role in DCM.

CircRNAs, a type of noncoding RNAs that function to regulate gene expression, can become key regulators of a variety of pathological processes. Previous studies have shown that CircHIPK3 not only acts as a sponge of miR‐29b‐3p to inhibit its expression and upregulates that of downstream target genes, such as Col1a1 and Col3a1, thereby improving myocardial fibrosis in DCM through competitive endogenous RNA (ceRNA) mechanism, but also exerts a ceRNA mechanism by acting as a sponge of miR‐30a to block its function. Consequently, this increases endothelial proliferation and vascular dysfunction in diabetic retinopathy.^21^, ^22^ In DCM, CACR has been shown to exert a ceRNA mechanism and act as a sponge for miR‐214‐3p, chelating and inhibiting its expression to reduce its inhibition of caspase‐1 expression, and ultimately regulate the scorch death of myocardial cells.^14^ Among circRNAs derived from the MAP3K5 gene, circMAP3K5 was found to form a feedback loop with ten‐eleven translocation family member 2 (TET2) and miR‐22‐3p, thereby exerting the ceRNA mechanism and regulating differentiation and intimal hyperplasia of vascular smooth muscle cells.^23^ Moreover, it has also been shown to antagonize phosphorylation and inactivation of Akt1‐induced apoptosis signal‐regulating kinase 1 (ASK1) by encoding a novel protein that competitively binds to the original protein, thereby activating ASK1‐induced apoptosis and reducing resistance to gefitinib.^24^ To date, however, specific mechanism underlying circMAP3K5 action in DCM remains unknown. Results of the present study first showed that circMAP3K5 was significantly upregulated in DCM, whereas silencing it suppressed apoptosis of cardiomyocytes in DCM. Overall, these results revealed that circMAP3K5 has a new function in myocardial injury under a HG environment, affirming that circRNAs play a crucial role in heart disease.

Apoptosis is not an autologous injury that occurs under pathological conditions but a death process in which cells actively strive to better adapt to the prevailing environment.^25^, ^26^ DAPK2, a protein that belongs to the serine/threonine protein kinase family, is reportedly involved in apoptosis translocation. Previous studies have shown that silencing of DAPK2 effectively suppresses the rate of apoptosis. As an upstream regulator of DAPK2, miR‐22‐3p does not only interact with noncoding RNA in a variety of cardiovascular diseases but also plays an important role in the development of DCM.^27^ Previous studies have demonstrated that the temporal pattern of circulating miR‐22‐3p is a powerful and independent prognostic factor in patients with stable congestive heart failure.^28^ In addition, miR‐22‐3p was found to inhibit fibrogenesis of Ang‐treated cardiac fibroblasts by targeting platelet‐activating factor receptors, indicating that it plays a crucial role in the fibrogenesis process.^29^, ^30^ Moreover, miR‐22‐3p can also be regulated by the upstream circMAP3K5, thereby affecting the expression of downstream TET2, and regulating differentiation as well as intimal hyperplasia of vascular smooth muscle cells.^23^ In DCM, Additional research evidences have shown that miR‐22‐3p has is regulated by the upstream lncRNA, MIAT, thereby affecting the expression of downstream DAPK2 and promoting apoptosis of cardiomyocytes in DCM.^27^ These findings corroborate our results, in which during DCM development, circMAP3K5 exerted the ceRNA mechanism as a sponge chelating agent of miR‐22‐3p to inhibit its expression, and downregulate and upregulate miR‐22‐3p and downstream DAPK2 expression, respectively. Eventually, these phenomena promote the rate of apoptosis in cardiomyocytes during DCM development.

In summary, our findings demonstrated that circMAP3K5 not only has potential as a starting regulator of miR‐22‐3p but also modulates expression of downstream mRNA DAPK2 and eventually regulates the rate of apoptosis in cardiomyocytes during DCM development. Therefore, circMAP3K5 has promise as a new therapeutic target for DCM.

## FUNDING INFORMATION

National Key R&D Programme of China (2016YFC1301402).

## DISCLOSURES

None.

## Supporting information


**Supplementary Table S1.** Polymerase chain reaction primer design.

**Supplementary Table S2.** The top 50 dysregulated circRNA ranked by fold changes in microarray data.
