# Supplementary material for: CircMAP3K5 promotes cardiomyocyte apoptosis in diabetic cardiomyopathy by regulating miR‐22‐3p/DAPK2 Axis
Source: J Diabetes. 2023 Sep 21;16(1):e13471. doi: 10.1111/1753-0407.13471 (PMC10809294; doi:10.1111/1753-0407.13471)
Supplement: Supplementary file 1 — Supplementary Table S1. Polymerase chain reaction primer design. [file JDB-16-e13471-s001.docx]

| Table 1 Polymerase chain reaction primer design | |
| --- | --- |
| **Gene** | **Bidirectional primer sequence** |
| GAPDH(rat) | F:5’ GCTCTCTGCTCCTCCCTGTTCTA3'  R:5’ TGGTAACCAGGCGTCCGATA3’ |
| rno-Map3k5_0005 | F:5' GAGCCAACACTACAGTCAGGAA3'  R :5’ TCAGGAGCTGAACGAAACG3’ |
| DAPK2(rat) | F:5’-TCCTGGATGGGGTGAACTAC-3’  R: 5′-CAGCTTGATGTGTGGAATGG-3′ |
| rno_U6 | F:5’-CTCGCTTCGGCAGCACA-3’  R:5’-AACGCTTCACGAATTTGCGT-3’ |
| rno-miR-22-3p | F:5’-GCCTGAAGCTGCCAGTTGA-3’  R:5’-GTGCAGGGTCCGAGGT-3’ |
| miR-22-3p  RT Primer | GTCGTATCCAGTGCAGGGTCCGAGGTATTCGCACTGGATACGACACAGTT |
