# Supplementary material for: CircMAP3K5 promotes cardiomyocyte apoptosis in diabetic cardiomyopathy by regulating miR‐22‐3p/DAPK2 Axis
Source: J Diabetes. 2023 Sep 21;16(1):e13471. doi: 10.1111/1753-0407.13471 (PMC10809294; doi:10.1111/1753-0407.13471)
Supplement: Supplementary file 2 — Supplementary Table S2. The top 50 dysregulated circRNA ranked by fold changes in microarray data. [file JDB-16-e13471-s002.docx]

| **circRNA** | **GeneSymbol** | **FC (abs)** | **Regulation** |
| --- | --- | --- | --- |
| rno_circRNA_009571 | Fkbp5 | 3.141347 | up |
| rno_circRNA_014008 | Nrxn1 | 3.127105 | up |
| rno_circRNA_000466 | RGD1306565 | 3.05978 | up |
| rno_circRNA_004268 | Glul | 3.001118 | up |
| rno_circRNA_004582 | Ehbp1 | 2.960186 | up |
| rno_circRNA_000473 | RGD1306565 | 2.882497 | up |
| rno_circRNA_013985 | Nrxn1 | 2.807464 | up |
| rno_circRNA_000461 | RGD1306565 | 2.759652 | up |
| rno_circRNA_005394 | Cadps | 2.740952 | up |
| rno_circRNA_000472 | RGD1306565 | 2.676635 | up |
| rno_circRNA_003127 | Cblb | 2.668372 | up |
| rno_circRNA_015152 | Anks1b | 2.663239 | up |
| rno_circRNA_012365 | Acer2 | 2.651187 | up |
| rno_circRNA_003395 | Klhl24 | 2.628834 | up |
| rno_circRNA_011752 | Plekha5 | 2.623876 | up |
| rno_circRNA_000463 | RGD1306565 | 2.563308 | up |
| rno_circRNA_000468 | RGD1306565 | 2.562584 | up |
| rno_circRNA_013989 | Nrxn1 | 2.559465 | up |
| rno_circRNA_000462 | RGD1306565 | 2.556291 | up |
| rno_circRNA_013991 | Nrxn1 | 2.548484 | up |
| rno_circRNA_013986 | Nrxn1 | 2.535556 | up |
| rno_circRNA_014002 | Nrxn1 | 2.526196 | up |
| rno_circRNA_012322 | RGD1560884 | 2.499429 | up |
| mmu_circRNA_44896 | Clstn2 | 2.487992 | up |
| rno_circRNA_013698 | Tmem63c | 2.473827 | up |
| rno_circRNA_011134 | Gpsm1 | 4.912761 | down |
| rno_circRNA_007259 | Dpysl3 | 4.004461 | down |
| rno_circRNA_013379 | Ptpn3 | 4.003262 | down |
| mmu_circRNA_31698 | Dpysl3 | 3.955043 | down |
| rno_circRNA_000964 | Fads2 | 3.329014 | down |
| rno_circRNA_009978 | Fmn1 | 3.178059 | down |
| rno_circRNA_006485 | Atxn1 | 3.06799 | down |
| mmu_circRNA_23123 | Rtn4 | 2.943024 | down |
| rno_circRNA_007790 | Nfix | 2.918936 | down |
| rno_circRNA_004393 | Atp1b1 | 2.816946 | down |
| rno_circRNA_009979 | Fmn1 | 2.768692 | down |
| rno_circRNA_007726 | Chd9 | 2.768298 | down |
| rno_circRNA_006487 | Atxn1 | 2.736876 | down |
| mmu_circRNA_18998 | Mtmr3 | 2.73245 | down |
| rno_circRNA_006490 | Atxn1 | 2.68936 | down |
| rno_circRNA_009981 | Fmn1 | 2.65595 | down |
| rno_circRNA_009983 | Fmn1 | 2.606871 | down |
| rno_circRNA_006494 | Atxn1 | 2.593313 | down |
| mmu_circRNA_32718 | Sh3pxd2a | 2.569597 | down |
| rno_circRNA_006491 | Atxn1 | 2.519554 | down |
| rno_circRNA_009982 | Fmn1 | 2.486626 | down |
| rno_circRNA_001651 | Calm3 | 2.476073 | down |
| mmu_circRNA_32359 | Vldlr | 2.475153 | down |
| rno_circRNA_005889 | Psd3 | 2.468784 | down |
| mmu_circRNA_45798 | Fgd1 | 2.460722 | down |
